# Task oriented training improves the balance outcome & reducing fall risk in diabetic population

**DOI:** 10.12669/pjms.324.10092

**Published:** 2016

**Authors:** Javeria Ghazal, Arshad Nawaz Malik, Imran Amjad

**Affiliations:** 1 Javeria Ghazal, Post Graduate Trainee, Pakistan Railway Hospital, Rawalpindi, Pakistan; 2 Dr. Arshad Nawaz Malik, Associate Professor, Riphah International University, Islamabad, Pakistan; 3 Dr. Imran Amjad, Assistant Professor, Riphah International University, Islamabad, Pakistan

**Keywords:** Berg balance test, Diabetes mellitus, Fall risk, Task oriented balance training

## Abstract

**Objectives:**

The objective was to determine the balance impairments and to compare task oriented versus traditional balance training in fall reduction among diabetic patients.

**Methods:**

The randomized control trial with descriptive survey and 196 diabetic patients were recruited to assess balance impairments through purposive sampling technique. Eighteen patients were randomly allocated into two groups; task oriented balance training group TOB (n=8) and traditional balance training group TBT (n=10). The inclusion criteria were 30-50 years age bracket and diagnosed cases of Diabetes Mellitus with neuropathy. The demographics were taken through standardized & valid assessment tools include Berg Balance Scale and Functional Reach Test. The measurements were obtained at baseline, after 04 and 08 weeks of training.

**Results:**

The mean age of the participants was 49 ±6.79. The result shows that 165(84%) were at moderate risk of fall and 31(15%) were at mild risk of fall among total 196 diabetic patients. There was significant improvement (p <0.05) in task oriented balance training group for dynamic balance, anticipatory balance and reactive balance after 8 weeks of training as compare to traditional balance training.

**Conclusion:**

Task oriented balance training is effective in improving the dynamic, anticipator and reactive balance. The task oriented training reduces the risk of falling through enhancing balance outcome.

## INTRODUCTION

Diabetes is considered a global disease and not restricted to any region nearly all countries in the world are challenging this chronic metabolic disease. Change in the way of living, quality of life, eating habits, lack of exercise and physical activity all play some role in increasing incidence of diabetes. International Diabetic Federation reports the number of diabetes is expected to rise from 2010 to 2030 as 54%, with 2.2% growth per year. It is two folds to the growth of world’s total adult population per annum.^1^ The prevalence of diabetes in Pakistan is almost 07 million people in 2015 and in city areas is 5.1% in males and 6.8% in females. Conversely occurrence of diabetes in males and females of rural areas is noted as 5 % and 4.8 % respectively. It also shows greater incidence of diabetes in urban women when compared to men.^2^

Gait and balance in diabetics are more affected and dysfunctional as compared to age matched healthy older adults and patients who have diabetic neuropathy exhibit severe impairment in balance.^3^ The gait dysfunction include the decreased cadence, shorter stride length and increase in stance time was observed with more difficulties on irregular surfaces.^4^ Postural control is integral part of mobility and it includes the static, dynamic, anticipatory and reactive feedback for appropriate ambulatory function. Reduced balance ability, decrease Proprioception and gait performance cause increase in the risk of fall in DM.^5^,^6^ The frequency of fall is more in diabetic population and showed the association of fall risk and diabetes.^7^ the level of confidence associated with balance is also compromised in elder population which directly affect the physical functioning.^8^

Balance training is a dynamic and complex form of exercises which is directly linked with reduction in fall risk and improves the quality of life. There are different regimes used for the appropriate training of balance and fall reduction.^9^ The simple conventional approaches mainly emphasise on the exercises which improves the generalized balance. The effectiveness of task oriented balance training program in enhancing balance and practice should be trained of controlling the center of mass regarding the base of support either it is stable or moving.^10^

The balance training program consists of exercises in different static and dynamic conditions. Weight shifting and balance practice is done in conditions like standing on single leg, double leg support, tandem standing, with additional head movement.^11^ The fear of fall is also one of main factor which reduces the level of mobility and participation restriction.^12^ The optimal goal in such training is to decline the fall risk and highlight the physiological functioning.^13^

The current study was planned to determine the frequency of balance impairment and also to compare the specific task oriented balance training in reducing fall risk as compare to conventional training.

## METHODS

The first part was descriptive survey and 196 patients were recruited through non probability purposive sampling technique. The diabetic patients were assessed through berg balance test for balance impairment. The second part was a randomized control trial. Total 18 participants were randomly allocated to two groups task oriented balance training group (n=8) and Traditional balance training group (n=10). The study was conducted in Pakistan Railway Hospital Rawalpindi from 1^st^ January to 30^th^ June 2015. Physician diagnosed cases of diabetes mellitus type II with neuropathy; 30-60 years age and either gender were included in the sample. Diabetic foot, cardiac problem, cognitive or speech impairment, genetic, inflammatory, musculoskeletal, infectious diseases, tumor and malignancy were excluded.

The written informed consent was taken and the study was approved from ethical committee of Riphah International University. The balance was assessed using standardized & valid assessment tools; Berg Balance Scale for dynamic balance assessment, functional reach test for anticipatory balance, rhomberg test for static balance and backward release test for reactive balance. Three reading were recorded against each scale. One at baseline before treatment, 2^nd^ after 04 weeks of treatment and 3^rd^ assessment was taken after 08 weeks of treatment. The SPSS 21 was used for statistical analysis and the t independent test was used to compare the mean for inferential analysis.

### Treatment protocol

Traditional Balance Training Group: the patients were given traditional balance training exercise for three days, 30 minutes per session for eight weeks. Task Oriented Balance training group: this group received task oriented balance training three days a weeks, session of 30 min per day for 8 weeks. Both cognitive and motor task were included in training. The following protocol was given for 30 minutes, three days a week for up to eight weeks. The therapists were there for support and to prevent from fall during the training.

## RESULTS

### Descriptive analysis: Balance Impairment

The mean age of patients was 49.73±6.79 and 66% were female in sample. The results depicts 57(29.1%) do walk regularly while 139(70.9%) don’t do walk. The descriptive analysis shows that 31(15.82%) of patients were having moderate risk of fall and 165(64.18%) of patients had low risk of fall at berg balance scale. 15(7.7%) patients having static balance impairment with mean 1.08±0.27, 169(86.2%) had dynamic balance impairment having mean of 47.10±7.7, and 177(90.3%) had decreased reactive balance with mean 2.42± 0.75. Only 129(65%) had decreased anticipatory balance with mean 8.56±3.18.

### Comparison between task oriented and traditional balance training

The result shows that 61% patients were females and the means age was 45.67 ±3.92.

Fig-I shows that the mean score of functional reach test at baseline for task oriented group was 20.25 ± 5.34, 19.60 ± 5.81 for traditional balance training group with (p value >0.05). This means that there was no significant difference in the two groups before treatment. The mean values for score of functional reach test after 4 weeks of exercise program is 26.88 ± 4.57 and 20.50 ± 5.82 for task oriented balance training group and traditional balance training group respectively and (p value <0.05) means that there was still no significant difference between groups. The mean value for function reach score of task oriented balance training group was 30.38 ± 1.77 and for traditional balance training group was 22.5±5.64 and (p value 0.001). Marked difference between the two groups at 8 week shows that task oriented balance training is more effective for anticipatory balance for balance training in diabetic neuropathy patients.

**Fig-1:**
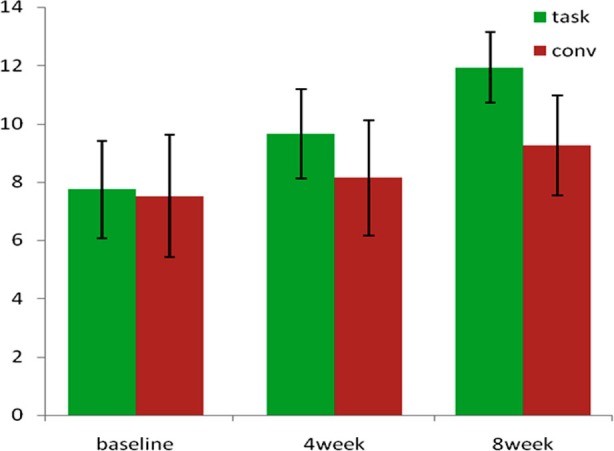
Shows the mean and SD of two groups.

This figure shows the functional reach test score difference between groups at baseline, after 4 weeks and after 8 weeks.

## DISCUSSION

This study observed the effect of task oriented balance exercise in improving types of balance in patients with diabetes. It showed that balance control has significant improvement using task oriented approach for balance training. Scores of berg balance scale suggested a remarkable improvement in dynamic balance between tasks oriented balance training group and traditional balance training group. There is a considerable difference in the outcome of anticipatory balance between the groups as recorded by functional reach test. The participants in this study practiced cognitive and motor tasks simultaneously in last four weeks of training. The results of this study suggested that dynamic and anticipatory balance is improved in task oriented training group than traditional balance training group.

**Table-I:** Inferential analysis for Berg Balance Scale.

Variable	Task oriented (Mean ± SD)	Traditional (Mean ± SD)	P value
Berg balance Scale at baseline	46.75± 2.12	47.60 ±1.84	0.37
Berg balance scale at 4 weeks	50.25 ±1.66	49.20 ±1.33	0.21
Berg balance scale at 8 weeks	54.88 ± 1.12	52.0± 3.36	0.02 *

The Table-I shows that there is significant difference in two groups. Hence the task oriented training is better than traditional balance training and 08 week training has significant contribution. (*p*=0.02*)

A case series was conducted by Sisupadol et al. in 2006 on balance training under single versus dual task setting in elderly having problems with postural instability. The patients who were given balance training in dual task setting demonstrated better improvement so these results are similar with the finding of this study.^14^ The study conducted by Geraldine L. Pellecchia in 2005 the subjects (n=18) were allocated to three groups viz; without treatment, dual task group and single task group. Single task group received postural task of static standing and cognitive tasks of counting backwards. In dual task group the subjects practiced both the tasks at the same time. It suggested that dual task ability improves with dual task training. The findings resembles with the outcome of this study as there was significant difference as reported in functional reach in task oriented and traditional group.^15^

This study further shows that scores on berg balance scale were improved after 8 weeks of the exercises. Sisupadol et al. conducted a double blind RCT in 2009 to evaluate the balance performance under the effect of single task against dual task balance training. The participants of the study were randomly divided into single task, dual task and dual task with variable priority groups. The score for Berg Balance Scale, Activity Balance Confidence were evaluated after four weeks. They concluded that single task and dual task training improves score on BBS and gait speed was also better in dual task group than single task training.^16^

The dynamic and anticipatory balance was assessed in diabetic neuropathic patients in this study and task oriented training and traditional training was applied to the patients for 08 weeks. The results found that there was marked difference in the score after the treatment session in both group and task oriented group. A Randomized control trial was carried out by Song et al. in 2011 and observed the outcomes of balance training on balance and proprioception in elderly. Participants were divided into two groups and Treatment group received and additional exercise program for balance for 60 minutes two times a week for 8 weeks, they reported increase in dynamic balance and improved scores were reported for the berg balance scale and Functional reach test. They suggested that balance training is good in rehabilitating the patients with diabetic neuropathy and balance impairment.^17^ Another study by Richardson et al. in 2001 determined the effects of exercise on balance on patients having diabetic neuropathy. They concluded that the exercises have good effect on the improvement of balance. Richardson et al also find out the level of confidence in DM with neuropathy patients after an exercise program. Balance in diabetic patients with neuropathy is reported to improve following a three week exercise program. This finding is also same as the finding of this study.^8^,^18^

This study assessed the risk of fall using berg balance scale and comparing the scores of berg balance scale at baseline and at the end of the training program. It is reported that decreased risk of fall was observed in the task oriented balance training group. In 2009 Donogue and stokes reported the minimal detectable difference in Berg Balance test varies as a function of the baseline score. They concluded that minimal detectable change is four points if baselines score of berg balance scale ranges from 45 to 56, five points for score from 35-44. If the score ranges 25-34, 7 points of difference is needed to detect the change. Five points increase is needed if the score 0-24. This study shows 8 points increase in mean score of berg balance scale at the end of task oriented balance training. So it is said that patients improved in balance and the risk of fall becomes better after task oriented balance training.^13^,^19^ A case report described the task oriented activities in stroke through the exer-gaming used to enhance the balance outcome. the task specific activities not only improves the functional status but also motivation and active engagement of the subjects.^20^ The task oriented training has significant effect on balance improvement and fall risk reduction because such approach has specific goal oriented and focus activities which directly linked with the performance of daily tasks. There were few limitation in the trial which include the small sample size, follow up constraint and non-cooperation from patients. Further studies should be done to explore the long term effect of training, cognitive effect and home based task activities.

## CONCLUSION

The study concludes that the balance impairment commonly affects the diabetic population. The diabetic patients have more at disadvantage to develop the increase risk of fall. The task oriented training for 08 weeks duration has better findings in improving the balance and decreasing fall risk. The proper and appropriate task oriented training must be included as part of diabetic management to prevent from further consequences and enhancing quality of life. Further research is needed to explore the other associated factor and other measures to improve social participation of diabetic population.
